# Our Penal and Prison System.—II

**Published:** 1922-10

**Authors:** 


### OUR PENAL AND PRISON SYSTEM.—II.

In our previous article we briefly traced the history of our present prison system. What is its condition and character now? These are described in great detail in the volume entitled "English Prisons To-day," to which we referred. The compilers of this book have collected a vast amount of evidence from many sources, giving, in most cases, the origin of their information. They have certainly established a case for further inquiry. They find many faults with the system. A number of these faults are matters of administrative detail. But others are inherent in the nature of the system itself. The view which we take of them will largely depend upon the view which we hold as to the object of punishment. The fatal error, which has so often been made in the past, has been to attempt amendment of the prison system without first examining the basis upon which it rests. What is the object which we attempt to achieve when we send people to prison? The question may appear to be a very simple one. But the variety of answers which we should find to be given by different people is sufficient demonstration that it is, actually, most complicated. The solution will largely depend upon our theory of punishment. So we must briefly consider what theories are held as to this.

Leaving aside the view that punishment is to be regarded as an expiation of religious sin, a view which is now antiquated, the theories of punishment can all be reduced to three, although attempts have been, and still are made to combine two or more of them.

1. We have the theory of Retribution. This was the original idea of punishment, and it is very ancient. Much scorn has been lavished upon this theory, and we are, at the present day, rather ashamed of it. But there can be no doubt that it forms the ultimate justification for penal measures in the minds of many people. As it appears to rest upon a fundamental instinct of mankind, it is doubtful whether we shall ever be wholly rid of it. And the most which we can do is to make it as little prominent as possible.

2. We have the Deterrent theory. This is, to many people, the true aim of punishment. From their speeches in court it appears to be the view which most judges and magistrates have in mind. On this theory it is held that the prospect of punishment may deter a man from committing crime, and that the recollection of punishment may produce a similar effect. We can have little doubt that in some, perhaps in many, cases this effect is produced. And, as R. M. McConnell has pointed out in his "Criminal Responsibility and Social Restraint," this deterrent theory of punishment is not incompatible with the strictest scientific determinism. The really difficult question is, why punishment sometimes does not deter. We see cases in which repeated punishments appear to have no deterrent effect, and others in which punishment appears to accentuate an anti-social attitude in the recipient. It seems quite possible to combine the deterrent aim with other measures.

3. Finally we have the Reformatory theory. This is often spoken of as if it were the only true object of punishment. And ideally, no doubt, it should be so. But we shall never attain this object merely by penal institutions of any kind. People often talk as if some form of institution was, *per se*, able to reform persons submitted to its methods. Institutions are sometimes reproached with their failure to reform. Reformation can, however, be, at most, indirectly due to any measures applied to a man.

The great fault in our penal system has always been that we have looked too exclusively at the particular act which a man has committed, and so have overlooked the man himself. We have measured out punishment in accordance with our estimation of the crime. But the crime is only a symptom. And no progress in criminology can be made until we are prepared to undertake a thorough study of causes. A necessary preliminary is the intensive study of the individual offender. We must examine him physically. It is true that criminologists no longer attach the same importance to certain physical "stigmata," as did Lombroso and his school. But physical defects may be of importance as incentives to delinquency, in so far as they affect the offender's mentality. Far more important is it to examine our offender from the mental side. This investigation must, of course, include the study of his intelligence, by means of mental tests, and otherwise. And much excellent work is being done in this direction. But our study must also include an investigation of the "unconscious" mind. The influence of this is only just beginning to be appreciated. But it is of the utmost moment, and throws a new light upon many points in criminality which have hitherto been most obscure. Without this complete and intensive study, punishment, which should really be regarded as in the nature of treatment, can only be applied in the dark. To deal with an offender without due examination is as unreasonable as to prescribe for a patient without examination. Mr. and Mrs. Webb lay down the principle that "the only justification for the imprisonment of unconvicted persons is the necessity of ensuring that they should not escape trial." We cannot agree. There is another, and a much more important object. At the present time, the only place in which the necessary examination can be made, in most cases, is the prison. And there are many reasons why it is most important that this examination should be made before trial. A full discussion of this question will be found in a paper by Dr. Hamblin Smith, of Birmingham Prison, in "The Journal of Mental Science," for July, 1922, which paper also contains an account of the work which is being done at Birmingham, with an estimation of its present results and possible future developments. The psychological examination of the individual offender is the clue to the solution of the problems of delinquency. If this necessary examination could be carried out elsewhere than in prison, it would, no doubt, be an advantage. But the facilities for this do not at present exist.

Both the books with which we are now dealing point out that an almost impenetrable veil of secrecy has been drawn over the prisons. Criticism appears to be resented. The prison officials were discouraged from giving, not merely information, but their views as to the system. And it would appear that the annual reports of the governors, chaplains, and medical officers are censored before publication. Indeed, since the war these reports have not been published at all. This secrecy seems to us to be unfortunate. Every system is the better for judicious criticism. And it is hard to see from what better source criticism could be obtained than from officials, many of whom have spent the whole of their working lives in the service. And the expression of the views of these officials would tend to correct some of the statements contained in the report of the committee, which have been, in many cases, derived from conscientious objectors, a class of prisoners who differed in many ways from the ordinary inmates of prisons. It seems, however, to be an invariable attribute of all government departments that they discourage criticism. The avoidance of controversy, of course, tends to ease of life. The administrators of any system always incline to satisfaction with what they administer, and the prison authorities are not exempt from this natural failing. In the days of local government, the justices were always quite satisfied with the state of their prisons, and resented any interference from the Home Office as unnecessary, subversive of discipline, and tending to "pamper" the prisoners. Major Arthur Griffiths, a prison inspector, actually wrote, in 1884, that the convict prisons were then "as perfect as they could be made." We do not suggest that the present Prison Commissioners are, in this respect, as bad as were their predecessors. There is, indeed, evidence that they are inclined to a more progressive policy. But it would appear as if changes were usually introduced on account of pressure from outside sources. It must be remembered that the Prison Commissioners are, ultimately, under the control of the Home Secretary, and that the Home Office is, to a large extent, managed by lawyers, and we need not pursue this point further.

It has been said that every judge and magistrate should be obliged to qualify for his office by serving a sentence of six months in prison. Without going so far as this, we think that it is unfortunate that judges and magistrates do not know more about the system to which they sentence offenders. Many of them do not take advantage of the right which they possess to inspect the prisons and to make themselves acquainted with the system in all its details. Criticism should be looked for and welcomed. Prisons should not, of course, be made a public show. But we think that much more first-hand knowledge of what goes on within their walls should be obtainable.

The great aim of prison administrators has always been to have, not only model prisons, but model prisoners. And by a model prisoner is meant, in this connection, one who obeys all orders and gives no trouble. The attainment of this end conduces to ease and smoothness of administration. But it is a fatal policy from the point of view of fitting the prisoner for life in society on release. The habit of simply obeying orders, and having all daily necessities supplied, is not one which in any way fits a man for ordinary life in society. And on release an inevitable reaction takes place.

Much of the committee's report is taken up with denunciation of the rule of silence, which is still officially in force in the prisons. It is no defence to say that this rule is largely evaded and ignored. It is this very evasion which has bad results, both upon prisoners and officers. There seems no good reason why conversation, so long as it is unobjectionable in itself, should not be allowed at proper times, care being taken to avoid contamination by means of classification. The possible danger of conspiracy will be urged against this. It is likely that this possibility is largely over-rated, and that it is a relic of the old days when the type of prisoner was wholly different to that of the present time. And there is much to be said for the contention that the officer should not merely supervise the prisoners at work, but should work with them.

Much stress is laid upon the alleged high rate of insanity, and of suicide and attempts thereat, among the prison population, as compared with the outside rates. It is an old controversy, and to enter into it fully would be a lengthy matter. But in estimating the value of the figures it must always be remembered that the prison population is, in many ways, abnormal, and that it consists, in the main, of persons between particular age limits. Also the average daily population of prisons is not a fair basis on which to ground comparisons.

We must give a few words to certain developments of our penal system which have been introduced in recent years. There is the system of probation. This is worked in different ways in various parts of the country. And to work it properly will certainly cause expense, although this expense, like all expenditure which assists to keep people out of prison, will be far more than repaid. Some authorities work the system in a most perfunctory manner. And those who make a genuine attempt to work it do not always get the proper officers, largely because they do not realise the nature of the problem. Healy and others have pointed out that all conduct, including, of course, delinquent conduct, is the direct result of mental life. As a consequence, the ideal probation officer must be a man or woman who has made a study of psychology, both theoretical and applied. It is not derogatory to the many excellent probation officers to say that they have not this knowledge. Indeed, in many cases, their very excellencies are a hindrance. Often they are required to report upon the home conditions of those under their care. Most probation officers regard as a perfect home one in which the parents are sober and attend church or chapel regularly. Now, any one who knows anything about the influence of home conditions upon children, influences which often have an enormous effect upon their character and conduct, is quite well aware that a home may reach this standard and yet be very far indeed from perfection. It seems incredible, but it is true, that a recent Government Commission upon the working of the probation system would appear to have taken no psychological evidence, and, in its report, ignored the medical and psychological aspect entirely.

Much has been heard of late about the Borstal system. This had its origin in a praiseworthy and honest attempt to overcome the appalling results of committing young boys and girls for long periods to prisons inhabited by adults. The Committee's report deals to a considerable degree with the Borstal institutions. It has praise for them. But it also points out defects. Young prisoners, as a rule, qualify for Borstal by a number of short sentences of imprisonment, and generally spend some time in a local prison before being sent to a Borstal institution. An arbitrary and indefensible limit of calendar age (16-21 years) is set up for the application of Borstal treatment, and this same fault is found in the arbitrary age assigned for the operation of the children's courts. The Borstal system is divorced from that of the Reformatory schools, which deals with offenders up to the age of 16 years, thus creating an artificial hiatus in the treatment of adolescent delinquency. The military element is too prominent in the system. And the report speaks of the medical officer as being incidental to an institution, whereas it should be obvious that the problem of adolescent delinquency is almost entirely a psychological one.

The third advance in our penal system is the establishment of the plan of "preventive detention." It is clear that there are some persons who cannot by any means be made to fit in with society, and who for their own sake, as well as that of society, must have some of their liberty restricted. Even Mr. Shaw is, to judge from his preface, willing to concede this. Preventive detention is a step in this direction. It was originally intended to be of an indeterminate character. It was, however, found that public opinion was not ready for this. At the present time, courts are, under certain conditions, allowed to add to a period of penal servitude a term of preventive detention of not less than five nor more than ten years. Thus the system is really of a penal character, and simply attempts to encourage longer sentences by permitting the judge to order that the latter part of the sentence should be served under less onerous conditions. If it were made possible to order a term of preventive detention apart from any preliminary penal sentence, it would mark a real advance in our system.

It has been long known that a considerable proportion of the inmates of our penal institutions were defective or subnormal mentally. How large this proportion is actually found to be will, of course, depend upon the standard of comparison which is adopted. Estimates vary, from the 60 per cent. (or even higher) claimed by some writers on the subject, to the 3 per cent. claimed by some experienced prison medical officers. We do not know at all what the average standard of mentality is for the whole of our population. But, judging from the investigations carried out upon the recruits of the American army, it is by no means high. And it is likely that the standard known among advocates of the "mental age" system as that of the "average adult" would not be attained by a very large percentage of our ordinary population. A good deal of work is now being done in this direction among our prisoners. From about 1897, "weak-minded" convicts have been collected at Parkhurst. And a few years later regulations were made for the local prisons, by which prisoners who, by reason of mental defect, were unfitted for ordinary penal discipline, were placed under the special charge of the medical officer, who was able, practically, to adopt the discipline to the prisoner. It was hoped that the Mental Deficiency Act would relieve prisons of a large proportion of such inmates. But its success in this direction has not been very great. The necessity of proving the existence of mental defect "from birth or from an early age" debars the Act from its full usefulness. There are not nearly enough institutions for the reception of these cases. And a number of local authorities are by no means anxious to apply the Act, largely on account of the expense involved. Still in progressive localities, in which cases are looked out for and segregated, the prisons have been largely relieved from, at least, the low grade cases. If the definition of a "moral imbecile," as now given in the Act, were amended by the omission of the words "some permanent mental defect," or if the nature of this defect were defined, the powers of segregation given by the Act could be employed for the elimination from prison of many habitual offenders.

It is, however, probably the case that by far the greater number of offenders present the problem of maladjustment rather than that of mental defect.

The position of the prison medical officer is of interest, and has undergone remarkable developments. From a position in which his main duty was to care for the bodily ills of such prisoners as were brought to his notice, he has advanced to a general supervisory power over the whole establishment. Under the present system it is not easy to see how his powers could be usefully increased. Further, there are indications that, in some districts, he is becoming the psychological adviser of the courts. He is undoubtedly hampered by the jealousy of science, which we mentioned before. And he is not over well treated, so far as the financial conditions of his service are concerned. He is also discouraged by the very slight notice which has, until recent years, been given to the research side of his work. It is, perhaps, a question whether his position is not injured by being an official of a penal department. And it might be an improvement if the medical side of prisons were taken over by the Ministry of Health. More co-ordination is certainly wanted between the prison, the asylum, and the mental deficiency services.

We have urged the paramount necessity for much more psychological examination of offenders. A demand for greater classification of prisoners naturally follows. Rational treatment is hampered by the way in which all kinds of prisoners are collected in our local prisons. It further follows that the abolition of short sentences is necessary. The manner in which courts serve out short sentences of imprisonment, with no reference to the character of the offender, and with no thought as to the possible effect of the sentence upon him, is unexplainable upon any rational view of punishment, and, in view of our recent advances in psychological knowledge, can only be described as scandalous. When prisoners can be properly classified, each could be assigned to the prison at which the treatment most appropriate to the conditions discovered in him could be applied. These ends can be achieved in no other way than by stimulating interest in the problem. This interest can best be stimulated by raising the veil of secrecy which has hung so long over our prisons, and also by adopting some form of local control. We have no wish to return to the old system of local government. But it should be possible to devise some means by which the administrative ability, which is to be found in many parts of the country, could be applied to the management of the prisons. This could be attained by giving the visiting committees much greater power than they at present possess. And, above all else, we must get rid of the fetish of a stereotyped uniformity, and we must also have power and encouragement to try experiments.

It will be noted that many of our suggestions involve expense. Many of the complaints in the committee's report could be obviated by an increase of staff. Some of our suggestions imply the existence of a properly paid staff, which would attract well qualified men and women. But expenditure of this kind is remunerative, and would be directly repaid, not to speak of the indirect saving, not only in money, but in human happiness. Once a community understands that the best way of preventing crime is, firstly, betterment of conditions in early life; secondly, careful study of the individual offender; thirdly, proper classification of offenders: and, lastly, an intelligent attempt to readjust the offending class, it will demand that its administrators should produce a plan which would revolutionise the present antiquated and most costly methods.

